# Identification of hub genes and diagnostic efficacy for triple-negative breast cancer through WGCNA and Mendelian randomization

**DOI:** 10.1007/s12672-024-00970-w

**Published:** 2024-04-12

**Authors:** Yilong Lin, Songsong Wang, Qingmo Yang

**Affiliations:** 1 Department of Breast Surgery, School of Medicine, The First Affiliated Hospital of Xiamen University, Xiamen University, Xiamen, 361003 Fujian China; 2 School of Medicine, Xiamen University, Xiamen, China

**Keywords:** TNBC, WGCNA, Breast cancer, Triple-negative breast cancer, Biomarker, CCNB1, Mendelian randomization

## Abstract

**Objective:**

Triple-negative breast cancer (TNBC) represents a particularly aggressive form of breast cancer with a poor prognosis due to a lack of targeted treatments resulting from limited a understanding of the underlying mechanisms. The aim of this study was the identification of hub genes for TNBC and assess their clinical applicability in predicting the disease.

**Methods:**

This study employed a combination of weighted gene co-expression network analysis (WGCNA) and differentially expressed genes (DEGs) to identify new susceptible modules and central genes in TNBC. The potential functional roles of the central genes were investigated using Kyoto Encyclopedia of Genes and Genomes (KEGG) and Gene Ontology (GO) analyses. Furthermore, a predictive model and ROC curve were developed to assess the diagnostic performance of the identified central genes. The correlation between CCNB1 and immune cells proportion was also investigated. At last, a Mendelian randomization (MR) analysis utilizing Genome-Wide Association Study (GWAS) data was analyzed to establish the causal effect of CCNB1 level on TNBC.

**Results:**

WGCNA was applied to determine gene co-expression maps and identify the most relevant module. Through a screening process, 1585 candidate hub genes were subsequently identified with WGCNA and DEGs. GO and KEGG function enrichment analysis indicated that these core genes were related to various biological processes, such as organelle fission, chromosome segregation, nuclear division, mitotic cell cycle phase transition, the cell cycle, amyotrophic lateral sclerosis, and motor proteins. Using STRING and Cytoscape, the top five genes with high degrees were identified as CDC2, CCNB1, CCNA2, TOP2A, and CCNB2. The nomogram model demonstrated good performance in predicting TNBC risk and was proven effective in diagnosis, as evidenced by the receiver operating characteristic (ROC) curve. Further investigation revealed a causal association between CCNB1 and immune cell infiltrates in TNBC. Survival analysis revealed high expression of the CCNB1 gene leads to poorer prognosis in TNBC patients. Additionally, analysis using inverse variance weighting revealed that CCNB1 was linked to a 2.8% higher risk of TNBC (OR: 1.028, 95% CI 1.002–1.055, p = 0.032).

**Conclusion:**

We established a co-expression network using the WGCNA methodology to detect pivotal genes associated with TNBC. This finding holds promise for advancing the creation of pre-symptomatic diagnostic tools and deepening our comprehension of the pathogenic mechanisms involved in TNBC risk genes.

**Supplementary Information:**

The online version contains supplementary material available at 10.1007/s12672-024-00970-w.

## Introduction

It is estimated that in 2020, breast cancer in females surpassed lung cancer, becoming the primary cause of new cancer cases globally. The number of new cases reported was 2.3 million, and 685,000 fatalities occurred [1]. Breast cancer subtypes are characterized by the presence of hormone receptors (HR) and the human epidermal growth factor receptor 2 (HER2). These subtypes display varying therapeutic sensitivities and clinical prognoses [2]. Triple-negative breast cancer (TNBC) comprises 15–20% of total breast cancers and is known for its aggressive progress, high incidence of recurrence, and poor prognosis. TNBC is defined by the absence of HR expression as well as the absence of HER2 overexpression or amplification [3, 4]. Unlike other breast cancer subtypes that utilize therapeutic targets like ER or HER, TNBC currently lacks approved targeted treatments. As a consequence, systemic chemotherapy remains the accepted standard of care for patients with TNBC [3]. Due to the limited treatment options available for TNBC, it is crucial to urgently investigate new targets that can enhance the prognosis of this condition. The identification of effective target genes is vital to making targeted therapy for TNBC more feasible. Recently, researchers have used bioinformatics techniques, such as single-cell analysis and RNA-seq analysis, to explore the mechanisms underlying TNBC [5–7]. To gain a deeper understanding of the molecular mechanisms underlying TNBC, it is crucial to integrate bioinformatics approaches with Mendelian randomization for the exploration of TNBC-associated biomarkers. Weighted gene co-expression network analysis (WGCNA) is a method for detecting hub genes related to TNBC, but few studies have been done in this regard. Furthermore, there has been no application of Mendelian randomization to validate the results of transcriptome analysis in TNBC.

In oncology, microarray analysis is used for various clinical purposes, including molecular cancer classification, tumor response prediction, and prediction of prognosis [8]. Using the WGCNA algorithm, highly correlated genes are systematically integrated into multiple modules [9]. WGCNA is a powerful tool for discovering the relationship between genes and clinical phenotypes and has been used to identify cancer markers like gastric cancer [10] and ovarian cancer [11]. As a result, identification of the expression of the appropriate biomarkers for identification and therapeutic evaluation is crucial for understanding the mechanisms of diseases such as TNBC [12, 13]. It was the goal of this study to identify core genes, novel biomarkers, or possible mechanisms associated with TNBC.

An epidemiological method, Mendelian randomization (MR), can be used to reinforce causal inference by using instrumental variables from genetic variants of an exposure [14]. As genetic variants are distributed randomly at conception and, consequently, uncorrelated with significant confounders, this approach minimizes any residual confounding [15]. MR requires the selection of genetic variants that are highly related to the exposure under investigation. As alleles are inherited randomly, individuals are assigned to different levels of exposure dosage [16]. In this study, the hub gene, CCNB1, was examined with two samples of MR data to determine if it is associated with the risk of TNBC.

In this work, differentially expressed genes (DEGs) between normal ductal cells of the breast and TNBC were examined. Using WGCNA, the most relevant modules were identified and intersected with DEGs, leading to the discovery of five potential diagnostic biomarkers, namely CDC2, CCNB1, CCNA2, TOP2A, and CCNB2. These biomarkers have the potential to contribute to the investigation of the mechanism of TNBC and serve as targets for immune therapy. Additionally, the causal relationship between CCNB1 expression and TNBC was verified through Mendelian randomization.

## Materials and methods

### Data source

All measured genes expression and grouping information from this dataset can be gained from the Gene Expression Omnibus (GEO) database. This dataset (GSE38959) was counted with transcriptome microarray assays in the mammary ductal cells obtained from TNBC tissues by means of immunohistochemical staining (N = 30) and normal tissues (N = 13) [17].

### Identification of differential expressed genes

Initially, the dataset GSE38959 was read through R software version 4.2.1. The dataset underwent preprocessing for correction and data normalization. Following this, DEG analysis searching was conducted by means of the "limma" package between TNBC and normal samples, and the adjusted p-value and |log fold changes (FC)| were calculated for each gene. Genes that met the criteria, adjusted p-value < 0.05 and |logFC|≥ 1.0, were considered as DEGs. Expression levels were analyzed, and volcano diagram and DEGs expression heatmap were generated using the R packages "pheatmap" and "ggplot2".

### WGCNA analysis

The study employed a methodical procedure of WGCNA to construct a gene co-expression network specific to triple-negative breast cancer. The WGCNA approach is frequently used to identify highly synergistic genomes and possible markers through an assessment of the interrelationship between such genomes and their relationship to phenomena [9]. By evaluating the interaction between each module and the molecular mechanism of triple-negative breast cancer, the most prominent module was selected as the central gene chosen by WGCNA.

### Searching of candidate genes and gene function analysis

To gain insight into triple-negative breast pathogenesis, we generated intersections and Venn plots for the candidate hub genes of WGCNA and DEG. To comprehend the potential mechanisms underlying progression and pathogenesis, Gene Ontology (GO) and Kyoto Encyclopedia of Genes and Genomes (KEGG) pathway analysis were conducted using the "clusterProfiler" R package [18].

### Hub genes screening of protein–protein interaction (PPI)

Trough the STRING (https://string-db.org/) platform and Cytoscape (https://cytoscape.org/) software, molecular interaction as well as PPI networks was predicted and visualized. The first step involved the use of the Degree algorithm in Cytoscape (https://cytoscape.org/) to rank and score the significant genes in the PPI networks. In the next step, we focused on the top 50 proteins arranged by degree and drew the protein–protein interaction for further analysis. Furthermore, top 5 proteins arranged by degree were selected was hub genes.

### Construction of nomogram model

The "rms" package was utilized to construct a nomogram model for assessing the risk of TNBC [19]. The predictive power of the nomogram model was evaluated by Harrell's concordance index [20]. Additionally, the "DynNom" R package was employed to explore the dynamics of TNBC risks. In order to determine the diagnostic efficacy of the candidate genes, the "ROC" package was employed to construct the receiver operating characteristic (ROC) curve. The accuracy of the ROC curve was indicated by the area under the curve (AUC) classified as high (AUC ≥ 0.9) [21].

### Analysis of immune cell infiltration in TNBC

The involvement of immune cells in TNBC was investigated by evaluating the degree of infiltration of 22 immune cells using cibersort analysis based on DEGs [22]. Stacked diagram was used to investigate the proportion in different samples. Next, we filtered the samples that met the conditions by p-value (p < 0.05). We plotted heatmaps to explore the infiltration of 22 immunocytes in each sample and violin maps to explore differential immune cells between TNBC and normal breast tissue cells. We utilized the 'corrplot' package to create heatmaps visualizing the correlation between 22 types infiltration of immunocytes. Furthermore, we used the 'ggplot2' package to analyze the correlation between immune cells and CCNB1 gene expression to investigate its role in the development of immune cells in TNBC tissues.

### Independent dataset validation

We further validated our results by applying a consistent DEG selection method (|logFC|≥ 1.0, adjusted p-value < 0.05) to two additional independent external datasets (GSE45827 and GSE65194), included 41 TNBC specimens and 11 normal breast specimens respectively, and dataset used in this study. Venn diagrams were generated to compare the DEGs identified from the three datasets. Interestingly, we found that the CCNB1 gene was located at the intersection of the three datasets, indicating the robustness of our findings.

### Survival analysis

To assess the clinical outcome, the CCNB1 gene was subjected to the Kaplan–Meier (KM) plotter (https://kmplot.com/analysis/) [23]. The KM plotter mRNA breast cancer database was applied to evaluate the prognostic values of CCNB1 in TNBC patients. In this study, TNBC patients were selected based on ER-negative, PR-negative assessed by IHC and HER2 -negative assessed by array. Probes of genes were selected based on the “only JetSet best probe set”. We plotted KM survival curves for the three main survival outcomes, including recurrence-free survival (RFS), overall survival (OS), and distant metastasis-free survival (DMFS).

### Two-sample Mendelian randomization

All statistics of the study was utilized in the open database. The Genome-Wide Association Study (GWAS) data source of CCNB1 was attained from ieu open GWAS project. The GWAS of the phenotype “G2/mitotic-specific cyclin-B1” was obtained in this study, including 3,301 samples and 10,534,735 SNPs. GWAS summary statistics of TNBC were obtained from the Breast Association (BRAC) and Discovery, Biology and Risk of Inherited Variants in Breast Cancer Consortium (DRIVE) [24]. In this study, inverse variance weighted (IVW) estimates was applied for the main analysis, which combined the Wald ratio of each SNP on the outcome and obtained an overall causal estimate. The assumption that the genetic variant influences the outcome only through the exposure was assessed for potential violation due to horizontal pleiotropy. If such pleiotropy exists, it would lead to bias in the causal estimates. To address this, analytical approaches were employed. Heterogeneity of the analyses was estimated by means of Cochran's Q test and its corresponding p-value. Furthermore, several statistical tests were performed to detect potential bias and pleiotropy. These tests included MR-Egger, weighted median, MR-PRESSO, single SNP analysis, and leave-one-out analysis. The MR-Egger method was used to correct for potential pleiotropy and obtain consistent causal inference in the presence of invalid instrumental variables. On the other hand, the weighted median approach was employed if invalid instrumental variables contributed to at least half of the weight in the analyses [25, 26]. The MR-PRSSO method was used to identify the Outlier SNP and correct the results to avoid potential horizontal pleiotropy [27]. In order to visualize our results, plots, including forest plot, funnel plot, scatter plot, and leave-one-out plot, were made to describe the robustness of the causal estimates of the MR analyses.

## Results

### DEGs identification

**Fig. 1:**
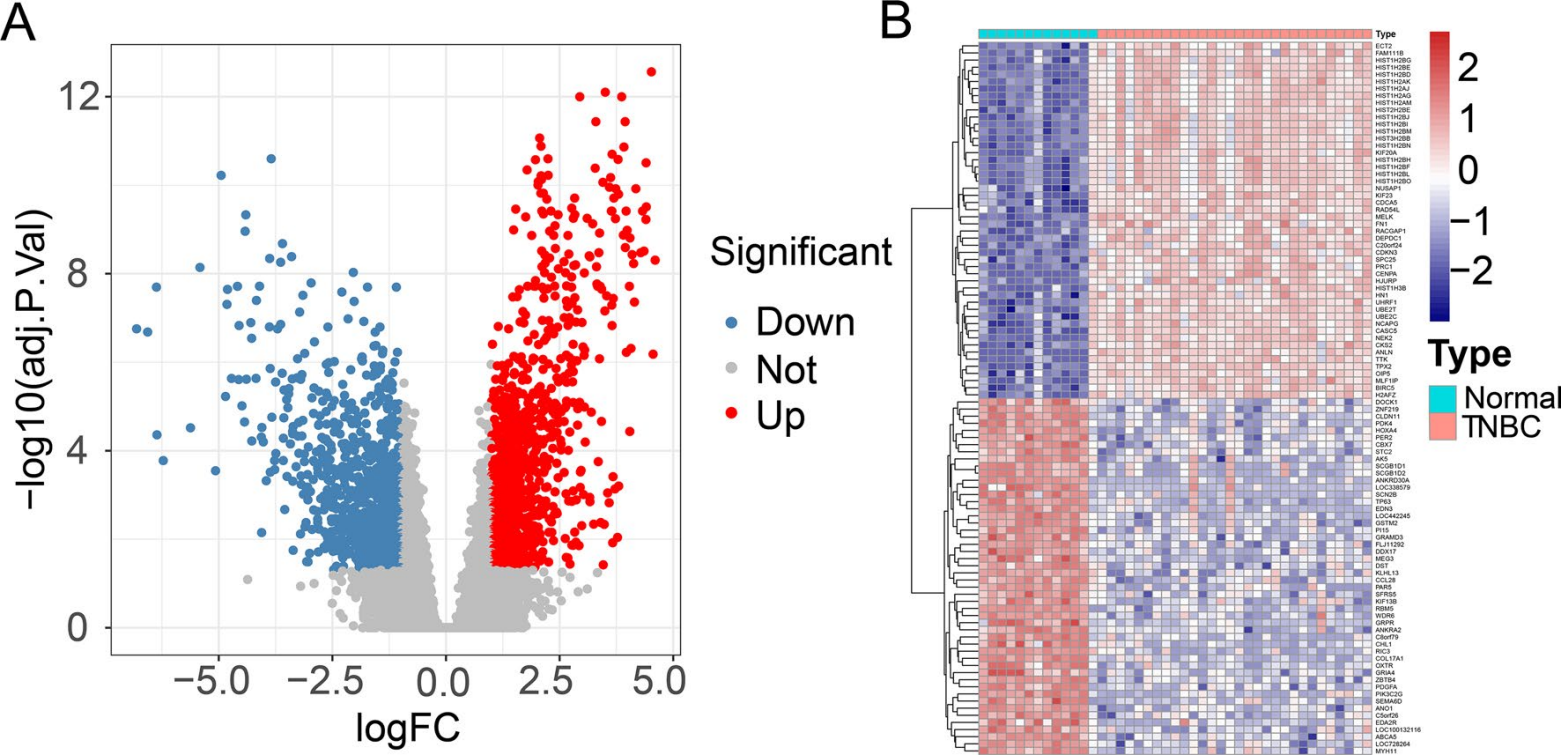
Genes differentially expressed between the TNBC and normal groups. **A** Volcanic map for differential expression analysis of GSE38959. **B** Heat map for differential expression analysis of GSE38959. Blue represents down-regulated genes, red represents up-regulated genes, and black represents undifferentiated genes

The TNBC dataset (GSE38959) was obtained from the GEO database and analyzed. By comparing the TNBC group with the normal group, 1850 DEGs were identified, consisting of 1004 upregulated genes and 846 downregulated genes (Fig. 1A, B; Supplementary Table S1).

### The identification of the TNBC-related module in the WGCNA network

**Fig. 2:**
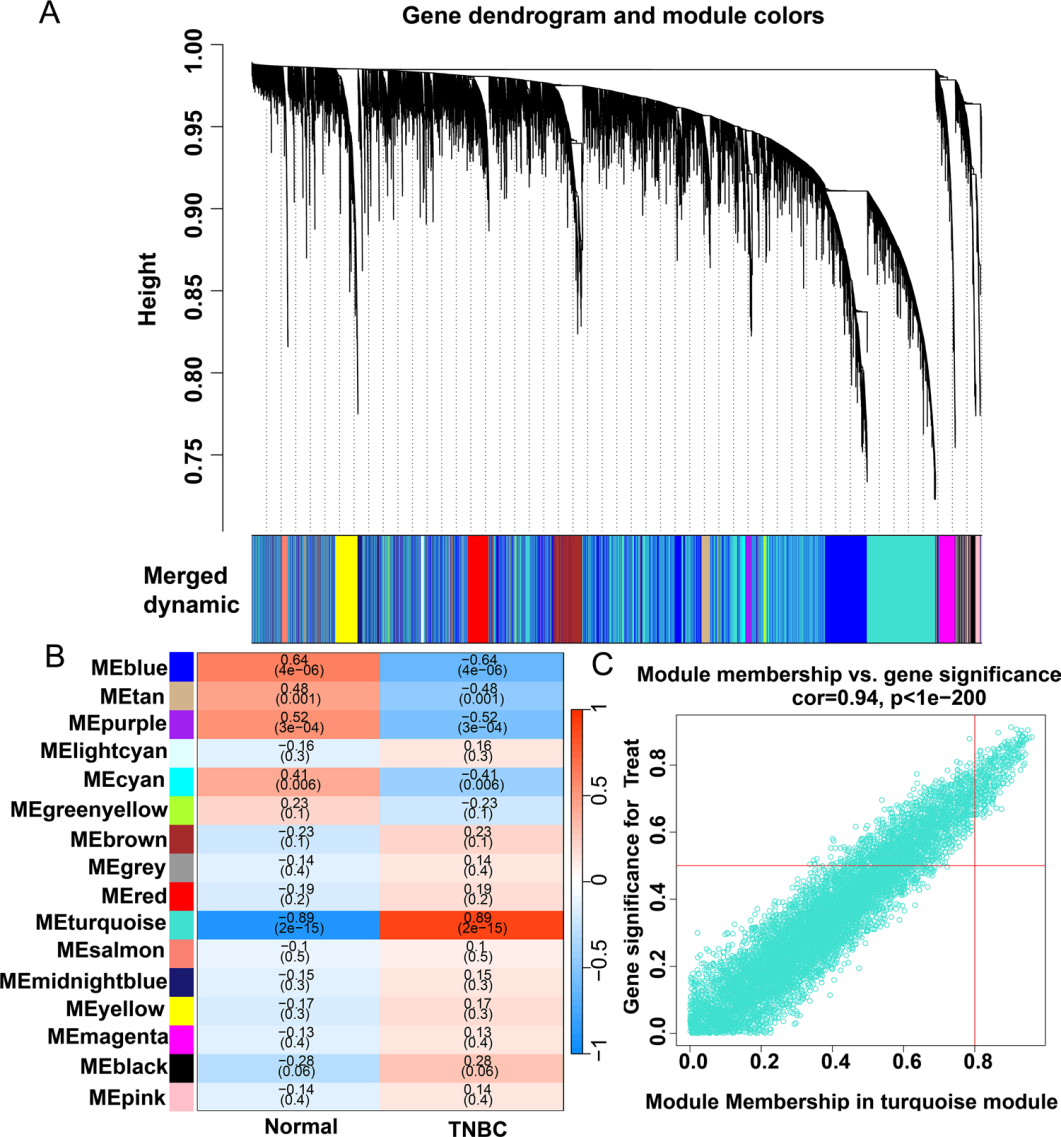
Identification of TNBC-associated gene modules in the GEO dataset using WGCNA. **A** The genes in the GSE38959 dataset were clustered into a dendrogram using a topological overlap matrix (1-TOM). Each branch in the dendrogram represents a gene, and co-expression modules were created in various colors. **B** Module-trait heatmap of the correlation between the clustering gene module and TNBC in the GSE38959 dataset. Each module contains the corresponding correlation coefficient and p-value.** C** Scatter plot of module turquoise has the strongest positive correlation with TNBC in the GSE38959 dataset

To explore the relationship between the potential gene modules and TNBC, we conducted WGCNA analysis on all candidate genes from the TNBC dataset (GSE38959) (Fig. 2A). Through this analysis, we identified 16 distinct modules (Fig. 2B). Subsequently, by analyzing the positive correlation coefficients, we were able to isolate the module turquoise from the GSE38959 dataset (Fig. 2C; Supplementary Table S2).

### GO/KEGG analyses

**Fig. 3:**
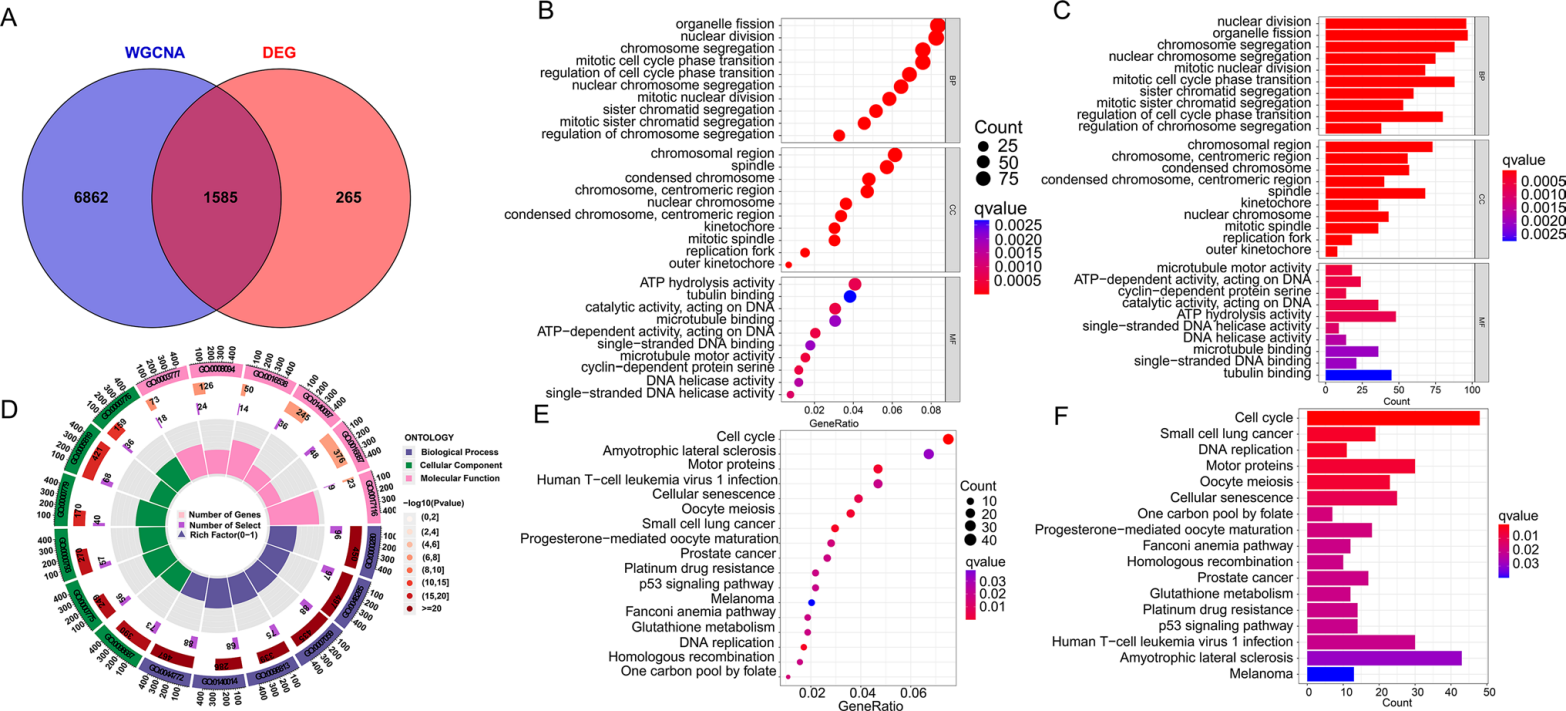
Candidate hub genes were screened and validated. **A** Venn diagram revealed 1585 overlapping candidate hub genes. **B, C, D** Enrichment analysis of candidate hub genes. **E, F** KEGG pathway analysis of candidate hub genes

By employing Venn diagrams, we identified 1585 overlapping genes as candidate hub genes, demonstrating potential significance in the progression of TNBC (Fig. 3A). To explore the co-expression of genes between the candidate hub genes derived from WGCNA and the DEGs, we conducted GO and KEGG analyses. The GO enrichment analysis revealed that these overlapping genes primarily impacted biological functions such as organelle fission, nuclear division, chromosome segregation, and mitotic cell cycle phase transition (Fig. 3B, C, D). Moreover, the KEGG enrichment analysis demonstrated the influence of these overlapping genes on cellular functions such as the cell cycle, amyotrophic lateral sclerosis, motor proteins, and cellular senescence (Fig. 3E, F).

### PPI network analysis

**Fig. 4:**
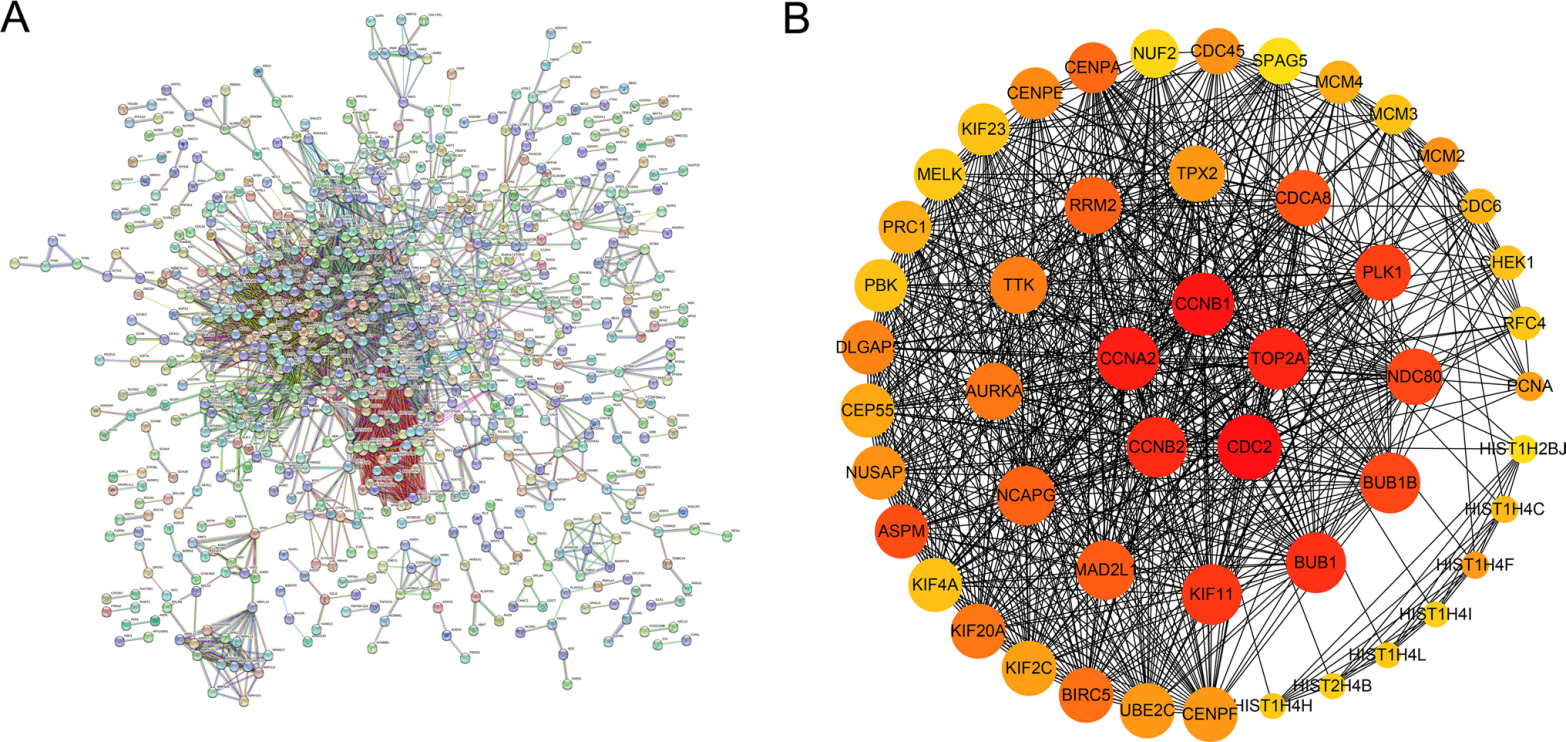
The construction of PPI network. **A** PPI network of overlapping candidate hub genes. **B** The top 50 protein of the interaction network were obtained by degree ssalgorithm

In order to create a protein–protein interaction network of hub genes, we utilized the STRING online tool. (Fig. 4A). The Degree algorithm in “Cytoscape” was used to rank and score the significant genes in the PPI networks. Protein interaction networks were mapped for the top 50 proteins to investigate potential mechanisms of TNBC development (Fig. 4B). Mainly, top5 proteins (CDC2, CCNB1, CCNA2, TOP2A, CCNB2) were selected was hub genes. The darker color of the circle stands for the higher score.

### Developing a nomogram model for predicting TNBC Risk

**Fig. 5:**
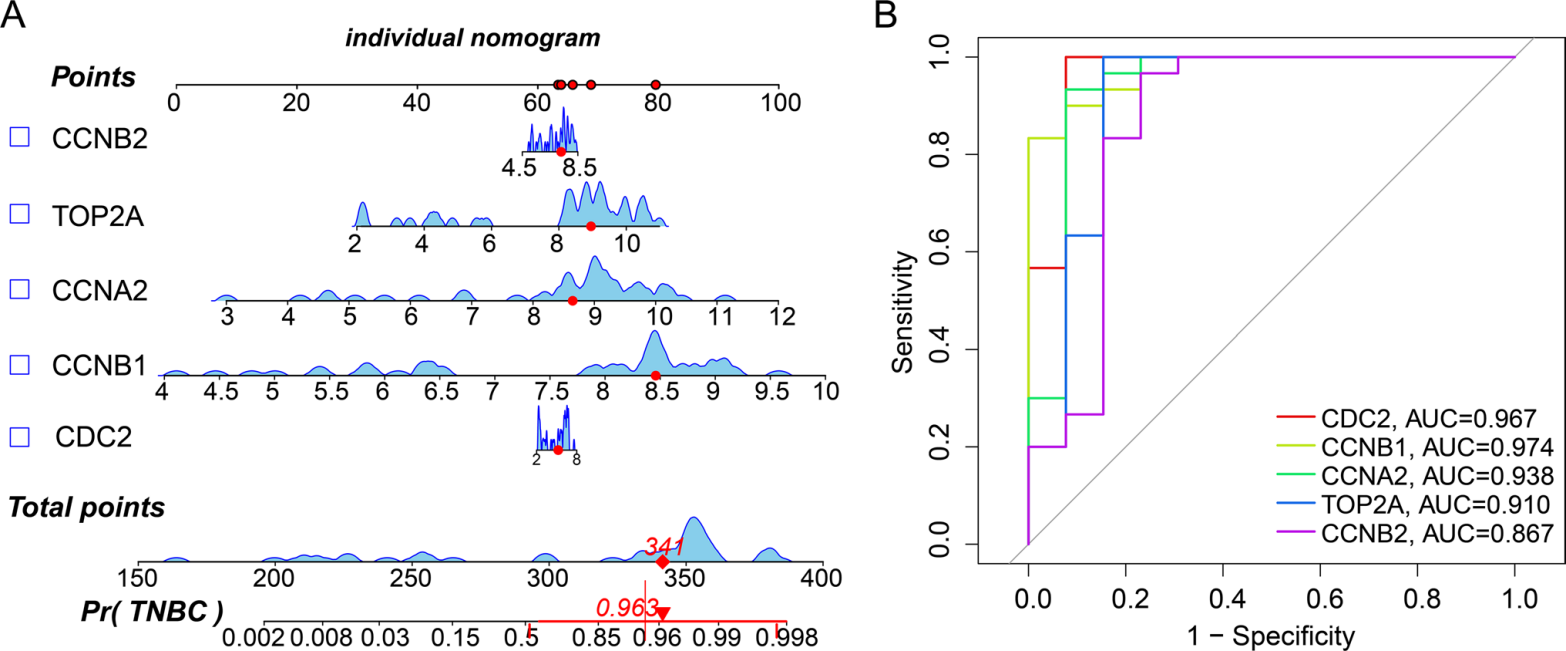
Predicting the risk of TNBC using nomograms. **A** Nomogram model of hub genes. **B** ROC curves to assess the diagnostic efficacy of nomogram model and each hub gene

A nomogram model was created to estimate TNBC risk (Fig. 5A). The "DynNom" R package was used to achieve the predict risk of TNBC with dynamic data. We then computed ROC curves of the five genes to investigate their diagnostic efficacy. The AUC of our nomogram model was also calculated to differentiate between TNBC and controls (Fig. 5B), demonstrating its effectiveness. The under areas of CDC2, CCNB1, CCNA2, TOP2A, and CCNB2 were 0.967, 0.974, 0.938, 0.910, and 0.867. Thus, our nomogram model accurately predicted the risk of TNBC, as demonstrated by the AUC values providing an accurate assessment of the diagnostic effect.

### Analysis of immune cell infiltration in TNBC

**Fig. 6:**
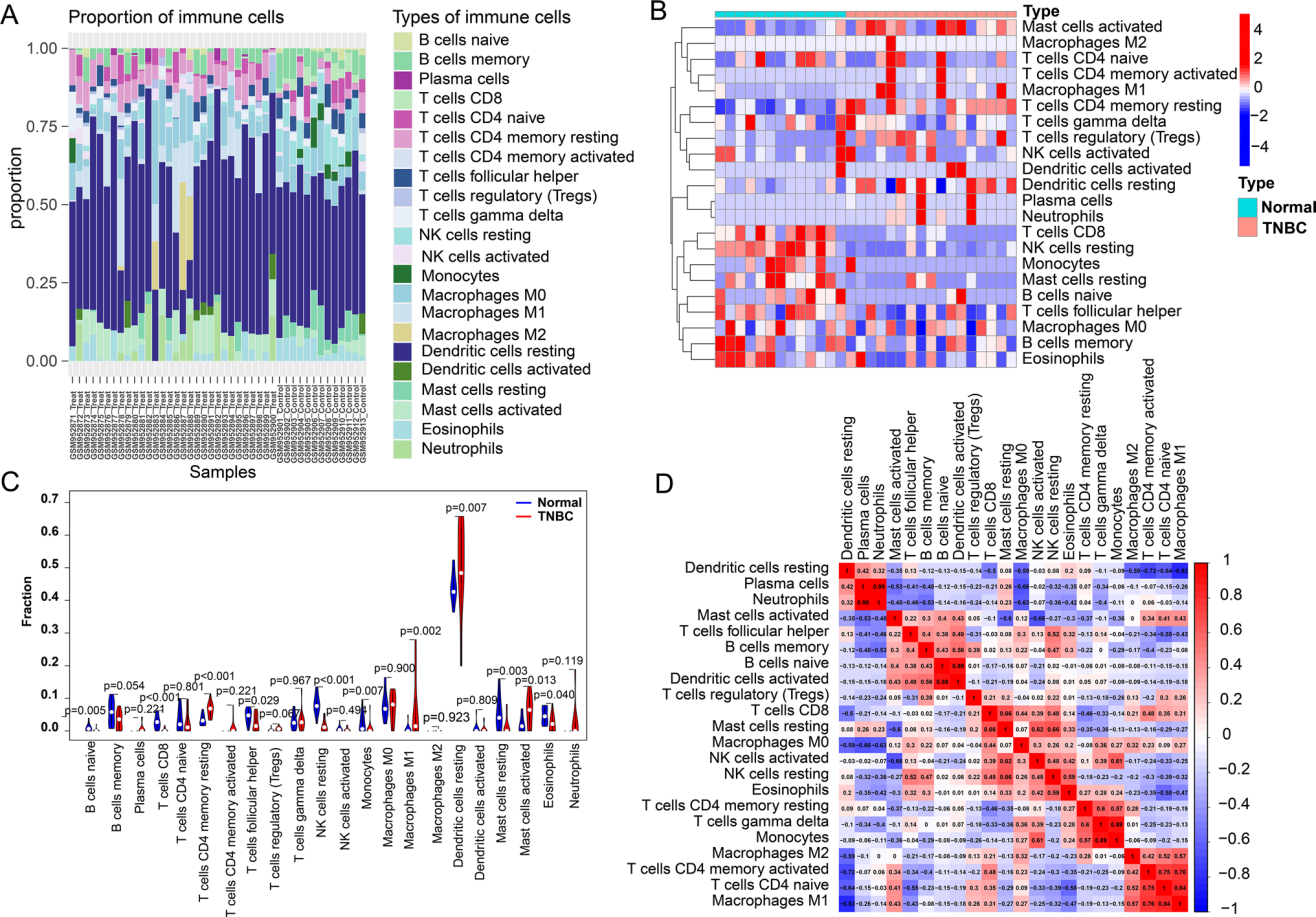
Immuno-correlation of CCNB1 in TNBC. **A** The immune cell infiltration proportion in different samples. **B** A heatmap of 22 immune cells in each sample. **C** The difference of immune cell infiltration between TNBC and normal groups. **D** The correlation of 22 immune cells

**Fig. 7:**
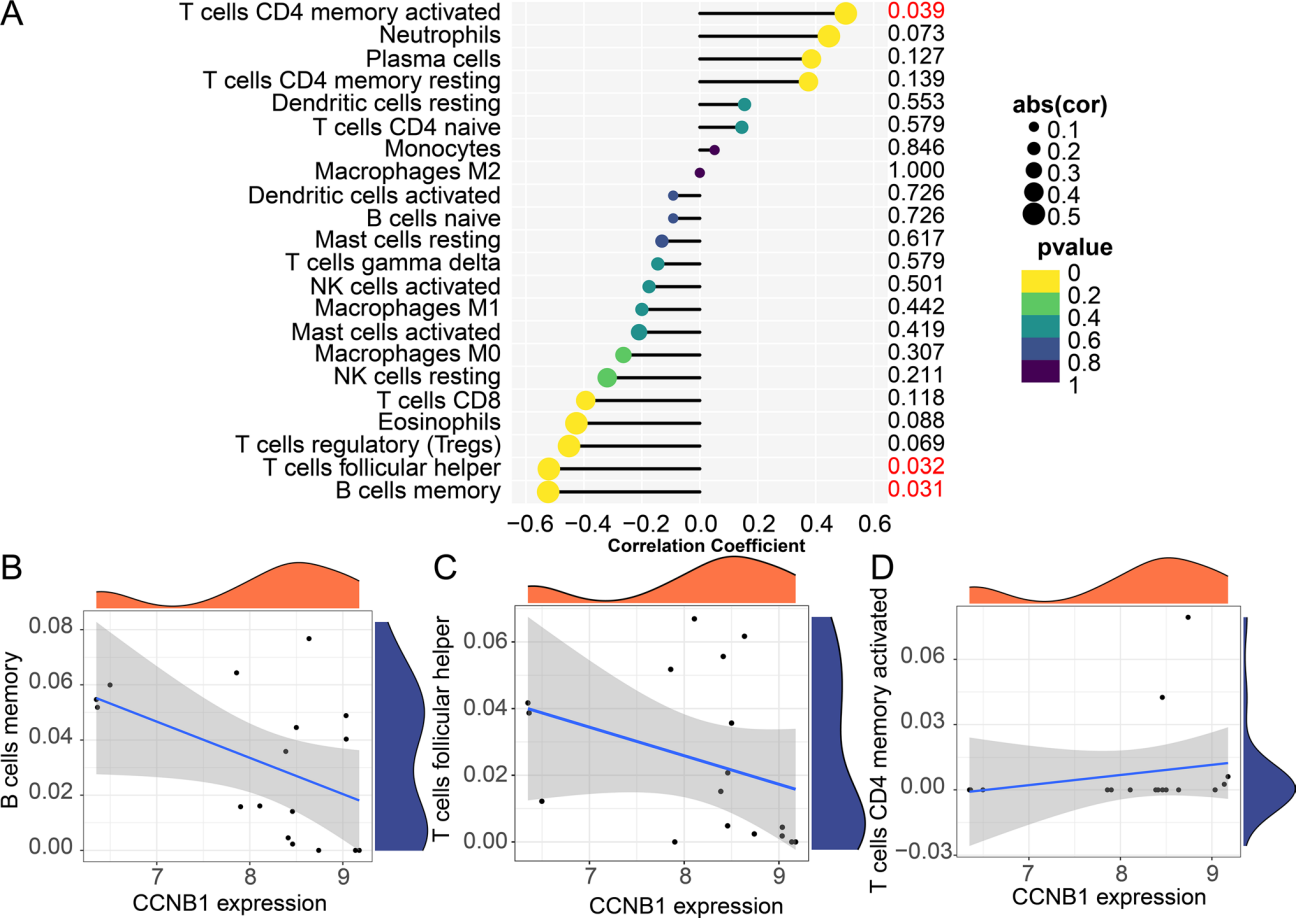
Correlation between CCNB1 and 22 immune cells. **A** The association between CCNB1 expression and memory B cells. **B** The association between CCNB1 expression and memory B cells. **C** The association between CCNB1 expression and follicular helper T cells. **D** The association between CCNB1 expression and activated memory CD4 T cells

For confirming the relationship between CCNB1 level and immune cells, the proportion of 22 immunocytes was analyzed using the ‘cibersort’ R package. A stacked diagram was drawn to show immune cell infiltration proportion in different samples (Fig. 6A). A heatmap of infiltration of 22 immunocytes in each sample (Fig. 6B) was plotted and violin plots indicated significant differences in 11 immune cells between TNBC samples and normal tissue (Fig. 6C). We also calculated the correlation between immune cells by correlation analysis (Fig. 6D) were identified. The correlation analysis between CCNB1 expression and immune cells revealed significant correlations with three immune cell types (Fig. 7A). Memory B cells (Fig. 7B) and follicular helper T cells (Fig. 7C) exhibited a negative correlation with the expression of CCNB1, whereas there was a favorable link between activated CD4 memory T cells and CCNB1 (Fig. 7D). This study provides further evidence supporting the hypothesis that immune cell activity and infiltration may be influenced by the level of the hub gene CCNB1.

### Independent dataset validation

**Fig. 8:**
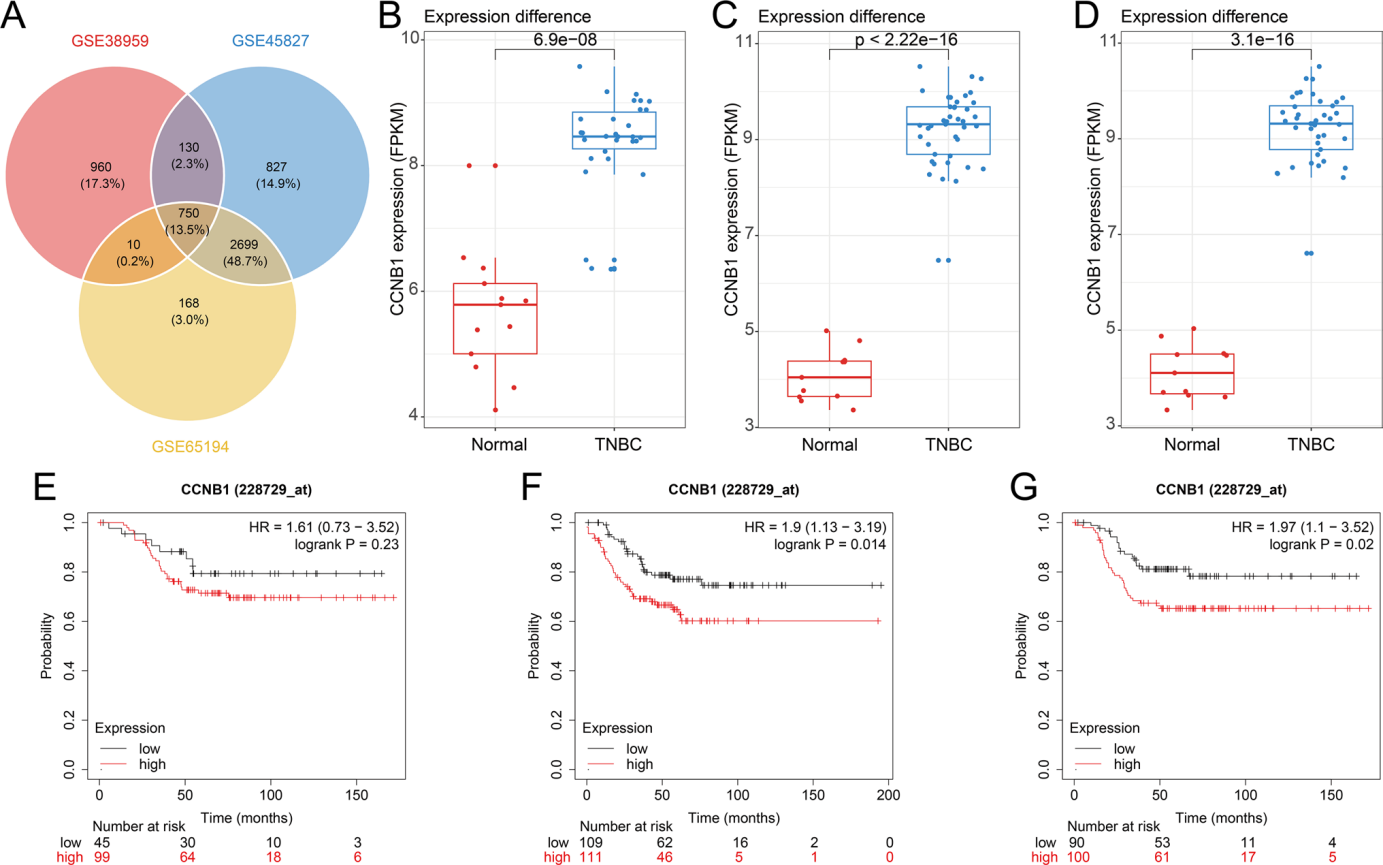
Independent dataset validation and survival analysis of CCNB1. **A** Venn plot of three independent dataset. **B** The CCNB1 expression (FPKM) difference between normal group and TNBC group in GSE38959. **C** The CCNB1 expression (FPKM) difference between normal group and TNBC group in GSE45827. **D** The CCNB1 expression (FPKM) difference between normal group and TNBC group in GSE65194. **E** OS analysis of CCNB1 in TNBC patients. **F** RFS analysis of CCNB1 in TNBC patients. **G** DMFS analysis of CCNB1 in TNBC patients

We obtained 4406 genes from GSE45827 and 3627 genes from GSE65194, respectively. Venn diagrams were generated to compare the DEGs identified from the three datasets (Fig. 8A). The expression of CCNB1 shows significant differences in each dataset (Fig. 8B–D). Interestingly, we found that the CCNB1 gene was located at the intersection of the three datasets, indicating the robustness of our findings.

### Survival analysis of CCNB1

To investigate the prognostic values of the CCNB1, the KM plotter bioinformatics analysis platform was used. We found that high expression of CCNB1 was associated with unfavorable overall survival of TNBC patients but it did not reach statistical significance (HR = 1.61; *P* = 0.23; n = 144) (Fig. 8E). While, overexpression of CCNB1 was an unfavorable prognostic factor of recurrence-free survival (HR = 1.90; 95% CI 1.13–3.19; *P* = 0.014; n = 220) (Fig. 8F) and distant metastasis-free survival in TNBC patients (HR = 1.97; 95% CI 1.10–3.52; *P* = 0.02; n = 190) (Fig. 8G).

### Causal relationship associated CCNB1 and the risk of TNBC

**Fig. 9:**
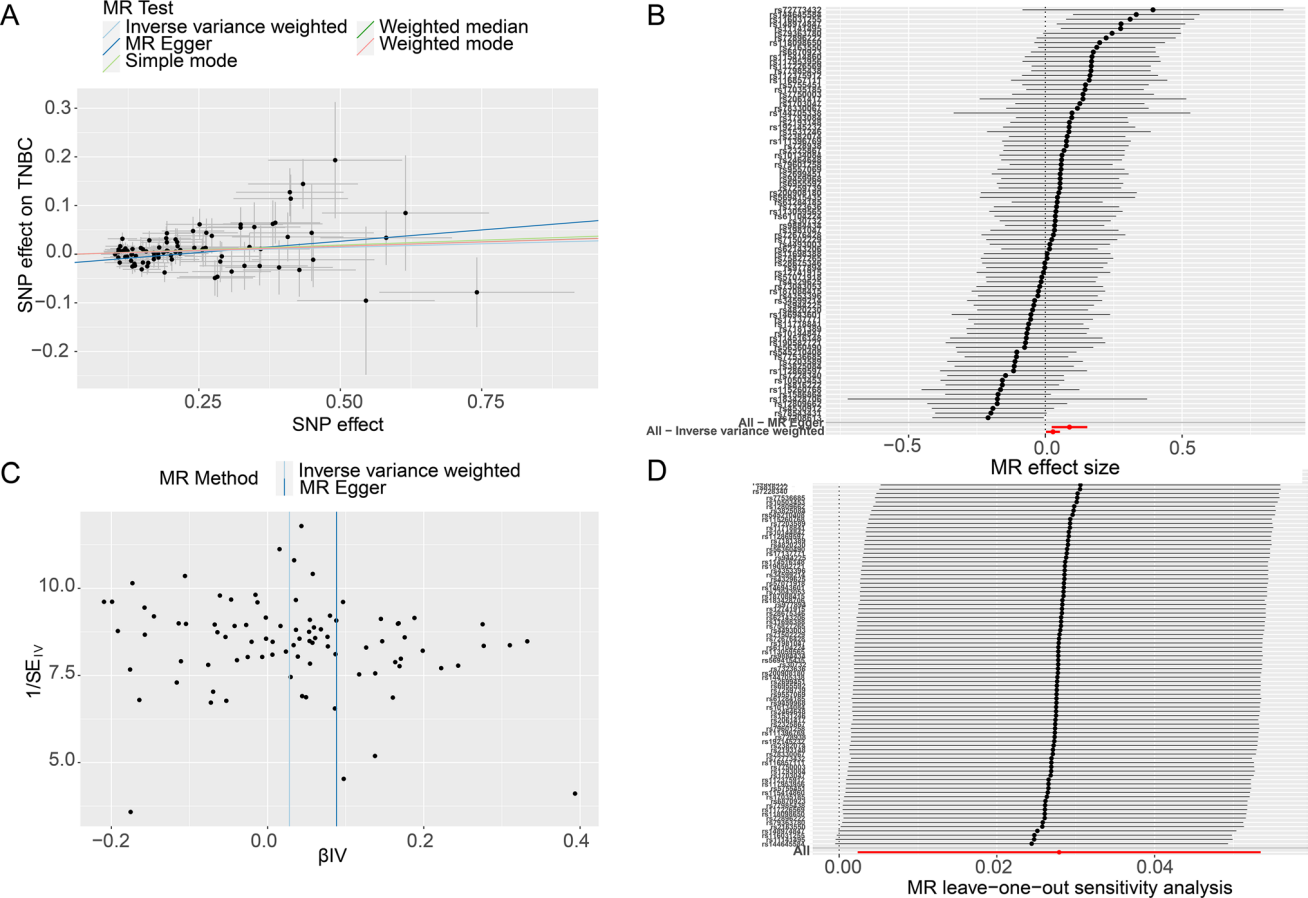
Mendelian randomization study results. **A** Scatter plot showing the causal effect of CCNB1 on the risk of TNBC. **B** Forest plot showing the causal effect of each SNP on the risk of TNBC. **C** Funnel plots to visualize overall heterogeneity of MR estimates for the effect of CCNB1 on TNBC. **D** Leave-one-out plot to visualize causal effect of CCNB1 on TNBC risk when leaving one SNP out

Supplementary Table S3 displayed the SNP characteristics of CCNB1 (P < 5*10^–5^). None of SNPs were considered weak instrumental variables. According to the three main assumptions of Mendelian randomization, the removal of five SNPs (rs215086, rs34383011, rs12198798, rs622354, rs117318310). The causal relationships of each genetic variation on TNBC were illustrated in Fig. 9A and B. Using the IVW method, we examined the causal relationship between CCNB1 and TNBC. The results revealed that each one-unit increase in log odds of CCNB1 led to a 2.8% higher risk of TNBC (OR: 1.028, 95% CI 1.002–1.055, p = 0.032). Additionally, significant statistical significance was observed with the MR–Egger method (OR = 1.092, 95% CI 1.024–1.166, p = 0.009) and the weighted median method (OR = 1.035, 95% CI 1.000–1.071, p = 0.049). As demonstrated in Fig. 9C, the funnel plot exhibited an approximate symmetrical causal effect. Moreover, there was no indication of heterogeneity, according to the MR Egger regression intercept (p = 0.369), suggesting that pleiotropy did not appear (p = 0.052). In Fig. 9D, we conducted a systematic MR analysis on the remaining SNPs after excluding every SNP individually, and the results remained significant. This demonstrates that all SNPs contributed significantly to the causality. Therefore, we can conclude that there was no dominant SNP in the relationship between CCNB1 levels and TNBC, validating the previous MR findings.

## Discussion

TNBC is a heterogeneous cancer from both biological and clinical perspectives, posing an unmet need due to its aggressive features and unfavorable prognosis [28]. Its chemoresistance, rapid invasion, atypical symptoms, and limited treatment options in clinical settings are major factors responsible for its poor outcome [29]. In this study, WGCNA and DEGs were used to obtain core genes, and we conducted analyses on immune infiltration and immune cell correlation. Our findings for the first time confirm the positive causal role of the CCNB1 gene in TNBC through Mendelian randomization.

Disease-related genes and biomarkers are valuable tools for detecting, diagnosing, prognosing, and monitoring therapeutic responses [30]. In a recent study, PPP1R14B was upregulated in TNBC tissues and correlated with paclitaxel resistance [31]. In breast carcinoma, TRPS1 was identified as a highly specific marker, particularly for TNBC based on TCGA database analysis and immunochemistry [32]. Another study identified four other genes as prognostic signatures for the disease-free interval by using DEG and PPI analysis [33]. Furthermore, through DEGs, WGCNA and PPI, our study discovered that the hub gene associated with TNBC was CCNB1, along with four other genes (CDC2, CCNA2, TOP2A, and CCNB2). The performance of our nomogram model in predicting triple-negative breast cancer was satisfactory, with CCNB1 being the most significant gene. By calculating the ROC curves, the efficacy of the five hub genes in distinguishing between TNBC and the normal group was assessed. The nomogram exhibited satisfactory AUC values, validating its potential as a reliable diagnostic tool. Importantly, CCNB1 demonstrated the highest discriminatory power. Therefore, it is essential to investigate the mechanism by which CCNB1 facilitates TNBC and increases its incidence.

CCNB1, one crucial molecule regulating the progression of the G2/M phase, is crucial for the cell cycle in mitosis [34]. Due to the significance of cell division and the cell cycle for tumor development, CCNB1 is crucial for tumor development. Overexpression of CCNB1 has been found in various tumors and is related to poor outcomes compared to the control group [35, 36]. CCNB1 expression is elevated in breast cancer tissue, and the expression of this biomarker demonstrates a significant correlation with patient survival time, tumor burden, methylation, infiltration of immune cells, as well as the absence of estrogen receptor expression [37]. Previous research has demonstrated notable links between CCNB1 and the absence of hormonal receptors, as well as the presence of HER2 receptors [38]. Additionally, CCNB1 has been related to TNBC in previous studies. Overexpression of CCNB1 is an unfavorable prognostic factor for TNBC patients compared to the normal group [39]. The decrease in cell viability at the G2/M phase in TNBC cells was observed upon the knockdown of PNO1, which was accompanied by the downregulation of CCNB1 and CDK1 protein expression [40]. Deregulated PNO1 also inhibited tumor growth in vivo and decreased the number and confluency of TNBC cells in vitro [40]. In this study, CCNB1 was found to be overexpressed in the TNBC group and exhibited strong performance in both the nomogram and the ROC curve. These findings align with those of previous research, thus further confirming our results. Our study provides additional evidence supporting CCNB1 as a promising therapeutic target for TNBC.

The involvement of immune cells in TNBC was investigated using cibersort's immune infiltration analysis in this study. A significant disparity in the expression patterns of diverse immune cell subsets was observed, including naive B cells, CD8 T cells, resting CD4 memory T cells, follicular helper T cells, resting NK cells, monocytes, macrophages M1, resting dendritic cells, mast cells resting, activated mast cells, and eosinophils. These findings are in line with previous research conducted in the field of cutaneous melanoma, which showed higher levels of activated CD4 + T cell infiltration in metastatic samples, indicating their potential contribution to cancer metastasis [41]. The CCNB1-specific CD4 T cell response has been studied insufficiently. However, T cell assay analysis demonstrated that CCNB1 has many CD4 T cell epitopes that are recognized differently by naive and memory CD4 T cells [42]. Notably, there was a positive correlation observed between CCNB1 expression in TNBC and activated CD4 memory T cells, while an inverse association was noted with T follicular helper cells and memory B cells. Furthermore, it has been shown that immune checkpoint therapy enables T follicular helper cells to enhance B immune cell activity, supporting the anti-tumor response [43]. The activation of B cells in T cells and the generation of antibodies play a vital role in the immune reaction. Therefore, these findings highlight the significance of tumor-infiltrating lymphocytes as a clinically relevant and reproducible biomarker that could impact the prognosis and treatment response of TNBC.

GWASs have significantly impacted the field of genetics in the last decade, particularly in complex disease research. They offer an unbiased method for exploring the genetic foundation of complex diseases [44]. The current investigation is the first to employ a two-sample MR analysis using numbers of GWASs to explore the causal relationship between CCNB1 levels and TNBC risk. MR is a comparable methodology to prospective randomized controlled trials (RCTs), which mitigates systematic biases impacting observational studies like confounding and reverse causality [45]. In this study, MR was creatively employed to authenticate the transcriptomics analysis findings. The findings suggested a possible causal link between serum CCNB1 levels and an increased risk of TNBC. In order to effectively minimize the regression dilution resulting from detection errors, highly accurate genotyping was used. To ensure the reliability of the findings, the MR-Egger regression test showed no indications of horizontal pleiotropy or heterogeneity.

Although this study revealed meaningful findings, certain limitations should not be ignored. Firstly, to increase the convincingness of the results, we should have included more TNBC datasets. Unfortunately, we were only able to analyze three datasets due to the lack of microarray data in the TNBC field. Secondly, although we employed bioinformatics analysis to examine the candidate hub genes and their potential functions related to TNBC development and used Mendelian randomization for validation, further biological experiments and clinical validation are necessary. These additional experiments and validations will help us confirm the exact mechanisms underlying the identified hub genes contributing to TNBC.

## Conclusion

We established a co-expression network using the WGCNA methodology to detect pivotal genes associated with TNBC. This finding holds promise for advancing the creation of pre-symptomatic diagnostic tools and deepening our comprehension of the pathogenic mechanisms involved in TNBC risk genes.

### Supplementary Information

**Supplementary Table S1**

**Supplementary Table S2**

**Supplementary Table S3**

## Data Availability

The datasets analyzed during the current study are available in the GEO database (https://www.ncbi.nlm.nih.gov/geo/). The GWAS of CCNB1 are available in the IEU OpenGWAS project (https://gwas.mrcieu.ac.uk/).

## Abbreviations

HR: Hormone receptors
HER2: Human epidermal growth factor receptor 2
TNBC: Triple-negative breast cancer
WGCNA: Weighted gene co-expression network analysis
MR: Mendelian randomization
DEGs: Differentially expressed genes
GEO: Gene expression omnibus
FC: Fold changes
GO: Gene ontology
KEGG: Kyoto encyclopedia of genes and genomes
PPI: Protein–protein interaction
ROC: Receiver operating characteristic
AUC: Area under the curve
KM: Kaplan–Meier
RFS: Recurrence-free survival
OS: Overall survival
DMFS: Distant metastasis-free survival
GWAS: Genome-wide association study
BRAC: Breast association
DRIVE: Discovery, biology and risk of inherited variants in breast cancer consortium
IVW: Inverse variance weighted
RCTs: Randomized controlled trials
